# Evaluating the effectiveness of digital health interventions for HIV prevention and treatment adherence among transgender populations: a systematic review and meta-analysis

**DOI:** 10.3389/fmicb.2025.1684834

**Published:** 2026-02-23

**Authors:** Alex Siu Wing Chan, Rayner Kay Jin Tan, Alston Choong, Kean Chang Phang, Sijia Li, Rongrong Wu, Patrick Ming Kuen Tang, Eleanor J. Ong, Jocelyn Cheung, Jakkrapatara Boonruang, Phoenix Kit Han Mo

**Affiliations:** 1 Department of Social Work, Hong Kong Baptist University, Hong Kong SAR, China; 2 Saw Swee Hock School of Public Health, National University of Singapore, Singapore, Singapore; 3 Department of Sports Medicine, Faculty of Medicine, University of Malaya, Kuala Lumpur, Malaysia; 4 Department of Pathology, Faculty of Medicine, University of Malaya, Kuala Lumpur, Malaysia; 5 Department of Anatomical and Cellular Pathology, State Key Laboratory of Translational Oncology, The Chinese University of Hong Kong, Hong Kong SAR, China; 6 Department of Health and Behavior Sciences, The University of Queensland, St Lucia, QLD, Australia; 7 Institute of HIV Research and Innovation, Bangkok, Thailand; 8 School of Public Health and Primary Care, The Chinese University of Hong Kong, Hong Kong SAR, China

**Keywords:** digital health interventions, HIV prevention, transgender populations, HIV testing, PrEP adherence, HIV care engagement

## Abstract

**Background:**

Transgender individuals face a disproportionate burden of HIV due to systemic barriers including stigma, discrimination, and limited access to gender-affirming healthcare. Digital health interventions (DHIs), such as mobile applications, telehealth, and online platforms, offer a promising avenue for delivering accessible, tailored, and potentially less stigmatizing HIV prevention and care services to this marginalized population.

**Objectives:**

This systematic review and meta-analysis aimed to evaluate the effectiveness of DHIs in improving HIV-related outcomes among transgender individuals. Specifically, it assessed the impact on HIV testing rates, care engagement, and stigma reduction.

**Methods:**

Following the Preferred Reporting Items for Systematic Reviews and Meta-Analyses (PRISMA) guidelines, a comprehensive search was conducted across five electronic databases—PubMed, Web of Science, Google Scholar, ScienceDirect, and Wiley Online Library—from January 2020 to March 2025. Eligible studies included randomized controlled trials (RCTs), quasi-experimental studies, and observational studies focusing on transgender populations and evaluating DHIs for HIV prevention or care. Screening, data extraction, and quality assessment were performed independently by two reviewers. A random-effects meta-analysis was conducted for quantitative synthesis, with heterogeneity assessed using *I*^2^ statistics. Subgroup analyses were performed by intervention type and year. Publication bias was evaluated using funnel plots and Egger's test.

**Results:**

A total of 11 studies were included in this meta-analysis. The pooled analysis demonstrated a significant positive effect of DHIs on HIV prevention and care outcomes, with an overall effect size (θ) of 1.82 (95% CI: 1.61–2.02). Heterogeneity was low (*I*^2^ = 13.32%), indicating consistent results across studies. Subgroup analyses confirmed sustained effectiveness across different years (2018–2024) and intervention types. No significant publication bias was detected (Egger's test, *p* = 0.147). The findings indicate that DHIs are acceptable and feasible, effectively improving key outcomes such as testing uptake and adherence.

**Conclusion:**

Digital health interventions are effective tools for enhancing HIV prevention and care engagement among transgender populations. Their success is likely attributable to increased accessibility, reduced stigma, and tailored design. To maximize impact and equity, future DHIs must be intentionally designed to be culturally competent, gender-affirming, and inclusive of the diverse spectrum of transgender identities, with ongoing research needed to evaluate long-term efficacy and scalability across diverse global settings.

**Systematic review registration:**

identifier: CRD420251180979.

## Background

Digital health interventions (DHIs) play a significant role in HIV prevention, especially among transgender individuals, who face disproportionate HIV risk due to factors such as stigma and limited access to healthcare services (Kuehn, 2021). These interventions, which include mobile applications, telemedicine, and online support, offer opportunities for tailored HIV prevention strategies that are more accessible and less stigmatizing. Empowering transgender women with gender-affirming digital health solutions can improve their engagement in preventive health behaviors (Kuehn, 2021; Budhwani et al., 2022; Jalil et al., 2023; Sheth et al., 2024). The integration of electronic health records into DHIs can further enhance the accessibility of preventive services (Huffstetler et al., 2022; Browne et al., 2024; Hawkes et al., 2023; Liu et al., 2024; Adedoja et al., 2024; Norena et al., 2022). These interventions are critical in reducing HIV transmission rates among marginalized groups, thereby contributing to overall community health improvements (Harris, 2024; Sevelius et al., 2020; Agulleiro et al., 2023; Kim et al., 2024; Cooney et al., 2023; Poteat et al., 2020; Sharfstein et al., 2022; Dubov et al., 2025; Wesp et al., 2021; Boonyapisit et al., 2025).

### Conceptual definition of digital health intervention (DHI)

A DHI refers to any intervention that is delivered or augmented through digital technologies such as mobile applications, online platforms, telemedicine, wearable devices, and artificial intelligence, with the primary goal of influencing health-related behaviors, managing disease, or altering health determinants (Wienert et al., 2022; Iyamu et al., 2021). These interventions may be designed to address health issues at the individual, clinical, or population level, and they often function within structured frameworks for classification and reporting to facilitate systematic evaluation and evidence-based practices (Wienert et al., 2022; Maaß et al., 2024). DHIs are inherently complex, meaning they consist of multiple interacting components that engage with the user's specific context, including their health status, technological environment, and social factors (Gamarel et al., 2020; Carra et al., 2025; Valente et al., 2023; Bekker et al., 2022; Jadwin-Cakmak et al., 2022; Baguso et al., 2021). These components can range from educational content to behavior-change prompts and can even involve real-time health monitoring. This complexity necessitates a theory-driven design, with clear attention to both engagement strategies and the measurement of user outcomes, ensuring that the intervention meets its objectives (Voorheis et al., 2023; Bruxvoort et al., 2023; Nejadghaderi et al., 2024; Sullivan et al., 2023; Chen et al., 2025; Escobar-Viera et al., 2021; Reisner et al., 2021).

### Transgender individuals and HIV care engagement

Transgender individuals face considerable challenges in accessing HIV care, often due to systemic barriers such as stigma and discrimination. Studies highlight that transgender women experience significantly higher HIV rates compared to the general population, underscoring the need for tailored interventions to improve HIV care engagement (Kuehn, 2021; Gandhi et al., 2023; Adimora et al., 2021). Barriers, such as the lack of access to gender-affirming healthcare, contribute to mental health issues, which further deter engagement in regular HIV care (Poteat et al., 2023). Social determinants of health, such as economic instability and lack of social support, exacerbate these challenges, hindering effective care (Hong et al., 2023; Hightow-Weidman et al., 2022; Kuhns et al., 2022; Zhang et al., 2024; Frisch et al., 2023; Gonzalez et al., 2024; Martinez et al., 2025). To address these issues, implementing community-based health initiatives and educational campaigns designed specifically for transgender individuals has been recommended (Kuehn, 2021; Poteat et al., 2023). In addition, integrating mental health services into HIV care can improve trust and retention in healthcare systems, addressing both mental health and chronic disease management (Poteat et al., 2023; Peck et al., 2024).

### ART and HIV care for transgender individuals

Transgender individuals face significant challenges in accessing HIV care, particularly due to systemic barriers to gender-affirming healthcare services. Research indicates that effective treatment protocols, such as antiretroviral therapy (ART) tailored to their needs, are crucial for managing HIV in this population (Zalla et al., 2023; Gandhi et al., 2023). In addition, policies promoting gender-affirming surgeries have been linked to improved mental health outcomes, as they decrease feelings of gender dysphoria and rates of depression, thereby supporting individuals' overall wellbeing (Schoenbrunner et al., 2023). Furthermore, studies demonstrate that enhanced healthcare access, combined with policies that affirm gender identity, is essential for addressing the elevated HIV rates among transgender individuals, thereby promoting health equity (Poteat et al., 2023; Santos et al., 2024).

### Digital health interventions and HIV testing

Digital health interventions are crucial for improving HIV testing rates among transgender individuals. DHIs, including text message reminders and tailored online resources, have proven effective in enhancing engagement with health services and increasing HIV testing rates (Lyles et al., 2021; Spinelli et al., 2024; Grinsztejn et al., 2023; Maksut et al., 2023; Santos et al., 2022; Patel et al., 2023; Mimiaga et al., 2021; Sevelius et al., 2021; Dearing et al., 2023; MacDonell et al., 2022). Telehealth, which became more widespread during the COVID-19 pandemic, offers an additional avenue for expanding access to HIV care. However, telehealth must be designed inclusively to avoid exacerbating existing inequalities (Brubaker et al., 2024; Talal et al., 2024). Ensuring that digital health tools are culturally competent and accessible is essential for promoting equity in HIV testing and treatment among transgender populations (Tuddenham et al., 2022).

### HIV prevention and transgender populations

Transgender populations face unique challenges in HIV prevention, primarily due to elevated infection rates and structural barriers (Ramasawmy et al., 2022; Mayer and Allan-Blitz, 2022; Clarke and Braun, 2017). Comprehensive strategies, such as the provision of preventive measures, are critical for reducing HIV transmission within this group. Research has demonstrated that tailored interventions can significantly reduce HIV transmission rates (Chou et al., 2023; Barry et al., 2023). Addressing social determinants of health and stigma through gender-affirming healthcare and community engagement enhances the uptake of preventive measures, improving health outcomes for transgender individuals (Chou et al., 2023; Gandhi et al., 2023; Adimora et al., 2021). These findings emphasize the need for inclusive health policies to combat disparities faced by transgender populations and support the broader fight against HIV (Gandhi et al., 2023; Liu et al., 2023).

### Digital health interventions and stigma reduction

DHIs such as internet-based storytelling, virtual reality (VR), and crowdsourced online platforms have proven effective in reducing stigma related to mental illness and other health conditions (Restar et al., 2023; Calabrese et al., 2022; Glynn et al., 2023; Kaur et al., 2025; Smith et al., 2025; Johnson et al., 2024). Interactive storytelling formats, for example, engage users more deeply than content-only approaches, fostering empathy and understanding (Fong and Mak, 2022). VR simulations have been shown to reduce stigma regarding dementia by providing immersive experiences for students and healthcare professionals (Ueno et al., 2023). Systematic reviews confirm that, although the stigma-reduction effects are generally small, they are consistent and influenced by delivery methods and context (Goh et al., 2021; Na et al., 2022). Crowdsourced platforms, such as online campaigns targeting hepatitis-related stigma, have also shown scalable success in reducing negative attitudes (Wong et al., 2022), with similar results observed in mental health stigma reduction through online interventions (Rodríguez-Rivas et al., 2022; Toth et al., 2023).

Digital health interventions also play a key role in reducing stigma associated with mental health and HIV care. Integrating DHIs into standard care can reduce self-stigma by fostering greater acceptance and improving access to services (Myers et al., 2022). For example, promoting mental health literacy through online platforms has been linked to reduced stigma among individuals with depressive symptoms, leading to better treatment engagement (Simon et al., 2024; Araya et al., 2021; Ramirez et al., 2024; Mayer et al., 2023; Gautam et al., 2024; Lee et al., 2024; Taylor et al., 2024; Aromataris and Munn, 2020; Cooney et al., 2024). The COVID-19 pandemic accelerated the adoption of digital mental health services, offering a timely opportunity to address stigma through increased support and engagement on digital platforms (Kola et al., 2021). These interventions not only support mental health but also reduce stigma within communities, ultimately improving wellbeing and health outcomes.

### Aim of the study

This systematic review and meta-analysis aimed to evaluate the effectiveness of DHIs in improving HIV prevention, care engagement, and stigma reduction among transgender individuals. Specifically, the study assessed the impact of DHIs on HIV testing rates, HIV care retention, and stigma reduction within this population.

### Research questions

How effective are digital health interventions in improving HIV testing rates, HIV care retention, and stigma reduction among transgender individuals?

## Methods

The study was conducted according to the Preferred Reporting Items for Systematic Reviews and Meta-Analyses (PRISMA) guidelines (Page et al., 2021), ensuring transparency and methodological rigor throughout the review process. The PROSPERO registration number is 1180979.

### Identification and selection of studies

A literature search was conducted to identify peer-reviewed articles reporting original research on digital health interventions (DHIs) for HIV prevention and care engagement among transgender individuals. The search, covering the period from January 2020 to March 2025, was carried out across the following electronic databases: Web of Science, PubMed, Google Scholar, ScienceDirect, and Wiley Online Library.

### Search strategy

Sensitive and specific search queries were generated with the help of relevant keywords and Boolean operators. The keywords included phrases related to digital health interventions, HIV prevention, care engagement, and transgender populations. Boolean operators (AND, OR) were employed to optimize search results. A sample Google Scholar search strategy included the following terms:

(“digital health” OR mHealth OR eHealth OR telehealth OR “mobile app” OR “online intervention” OR SMS OR telemedicine OR “social media intervention”) AND (“HIV prevention” OR “HIV testing” OR “antiretroviral therapy” OR ART OR “care engagement” OR “linkage to care” OR “adherence”) AND (transgender OR “transgender women” OR “transgender men” OR “gender diverse” OR “gender non-conforming” OR non-binary).

### Study selection

A total of two independent reviewers (A. S. W. C and J. C.) screened the titles and abstracts of retrieved records to identify potentially eligible studies. Full-text articles of potentially relevant studies were subsequently reviewed to determine final inclusion. Discrepancies during the screening or full-text review process were resolved through discussion with a third reviewer. All references were managed using Zotero version 6.0.36, which automatically identified retracted studies and flagged duplicate records. Duplicate entries were manually reviewed and merged by one of the reviewers.

### Eligibility criteria

#### Inclusion criteria

The inclusion criteria for this systematic review were based on the Population, Intervention, Comparison, Outcomes, Study Design (PICOS) framework (Methley et al., 2014). The population included transgender individuals (transgender women, transgender men, and non-binary/gender-diverse individuals) at risk for or living with HIV. The interventions included digital health interventions, such as mobile health (mHealth) applications, SMS reminders, telehealth services, online counseling, and social media-based interventions aimed at HIV prevention or care engagement. Comparisons were made with standard care or other relevant interventions. Primary outcomes included HIV testing uptake, ART adherence, linkage to care, and retention in care. Secondary outcomes included the acceptability, feasibility, and satisfaction with the interventions. Eligible study designs included randomized controlled trials (RCTs), quasi-experimental studies, cohort studies, and cross-sectional studies. Only studies published in English were considered.

#### Exclusion criteria

The following were excluded from the review: Non-original research articles, such as letters, editorials, and opinion pieces; conference abstracts, study protocols, and reviews; studies not published in English; and studies for which full texts were unavailable.

### Data extraction

Data from the selected studies were extracted using a pre-designed Microsoft Excel 2019 table. The first reviewer performed extraction, and the accuracy and completeness of the data were independently verified by the second reviewer. The extracted data included study identification details (authors, year), study design and setting, sample characteristics, intervention characteristics, outcome measures, and main findings. Primary outcomes were converted to standardized mean differences (SMDs/Hedges g) or risk ratios (RRs). Studies reporting only drug concentration or electronic monitoring data were converted to SMDs using formulas from the Cochrane Handbook. A random-effects model (DerSimonian-Laird) was used to pool effect sizes, with θ representing the overall SMD.

### Data analysis

#### Quantitative synthesis (meta-analysis)

A meta-analysis was conducted using Review Manager (RevMan, version 5.4.1) for studies with sufficient quantitative data. Dichotomous outcomes were analyzed using the Mantel–Haenszel method with a random-effects model, and the results were reported as odds ratios (ORs) with corresponding 95% confidence intervals (CIs). Furthermore, two primary outcomes were examined: HIV testing rates and experiences of condomless anal sex, comparing baseline (control) and follow-up (experimental) periods following digital health interventions.

#### Heterogeneity and sensitivity analysis

Heterogeneity was assessed using the Q statistic, *I*^2^, and τ^2^. Sensitivity analysis was conducted if *I*^2^ exceeded 50%. Publication bias was evaluated using funnel plots, Egger's regression test, and the trim-and-fill method.

#### Subgroup analyses

Pre-specified subgroup analyses were based on the following factors: year (2020–2025), intervention type (SMS, mobile applications, or telemedicine), geographic region (North America, Latin America, Asia-Pacific, Africa), and gender identity (transfeminine vs. transmasculine).

### Statistical methods

All statistical analyses were conducted using Stata 17.0 (metan, metareg) and R 4.3.1 (meta, meta for packages), with a two-sided alpha level of 0.05.

### Evidence certainty

The GRADE framework was used to assess the certainty of the evidence. Downgrading factors included risk of bias, imprecision, and potential publication bias.

### Ethical considerations

Ethical approval was not required as only published, de-identified data were analyzed.

## Results

### Study selection

**Figure 1:**
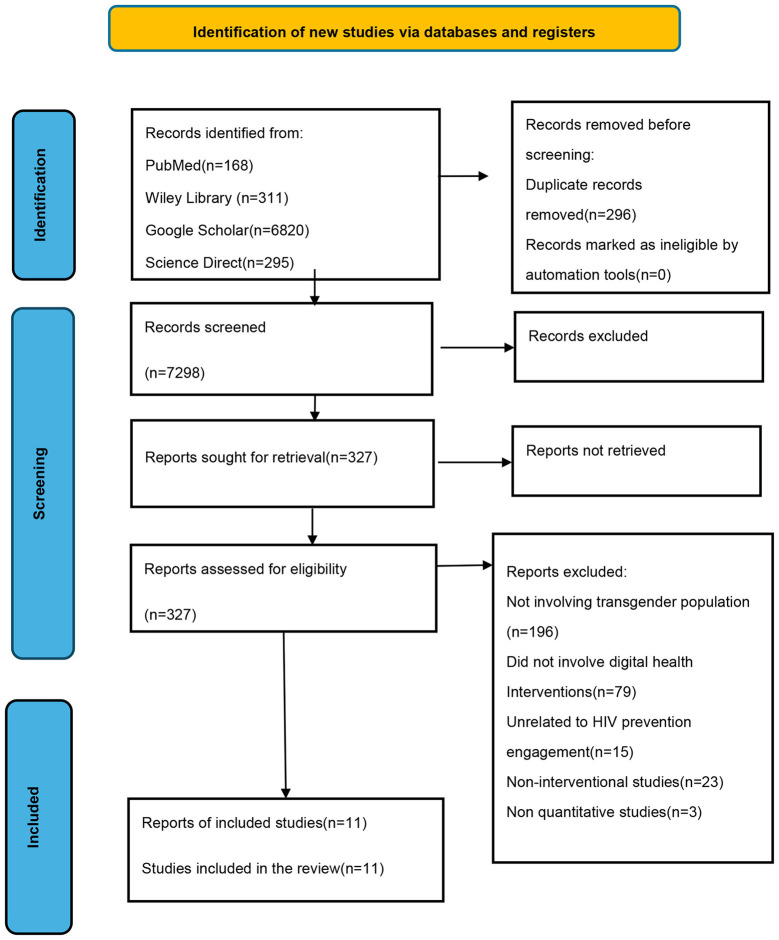
PRISMA flow diagram.

After independent review by two reviewers and subsequent verification, the meta-analysis included 11 studies published between 2018 and 2024, all of which investigated the impact of digital health interventions on HIV prevention and treatment adherence among transgender populations. The effect sizes for these studies ranged from 1.4 to 2.8, with most exceeding 1, indicating generally positive outcomes across studies. The results are presented in Figure 1 in the PRISMA flow diagram (Page et al., 2021).

The pooled effect size (θ) for the analysis was 1.817, with a 95% confidence interval of 1.613–2.021, suggesting a significant positive effect of digital health interventions on the targeted outcomes. The study by Magnus et al. (2018) reported the highest effect size of 2.0, while Kuhns et al. (2021) reported the largest confidence interval, ranging from 1.491 to 4.109. In contrast, some studies, such as Sun et al. (2020) and Schnall et al. (2024), reported effect sizes around 1.4, which are still consistent with positive effects but indicate slightly smaller improvements.

The 95% confidence interval for the majority of studies did not include 1, reflecting statistically significant positive effects. For example, the study by Stephenson et al. (2019) reported a confidence interval ranging from 1.31 to 2.29, indicating a clear positive effect. However, some studies, such as Garg et al. (2020), had a wider confidence interval (0.895–2.305), which could suggest variability in the data. The weight of each study ranged from 2.35% to 14.63%, based on the size and quality of the studies, with higher-weight studies such as Reback et al. (2021) and Stephenson et al. (2019) contributing more substantially to the overall pooled estimate (Table 1).

**Table 1:** Results of the meta-analysis.

**References**	**Effect size**	**[95% Conf. interval]**		**% Weight**	**Study quality**
Magnus et al. (2018)	2	1.456	2.544	11.68	High
Stephenson et al. (2019)	1.8	1.31	2.29	13.87	High
Sun et al. (2020)	1.4	0.805	1.995	10.05	High
Garg et al. (2020)	1.6	0.895	2.305	7.46	High
Kuhns et al. (2021)	2.8	1.491	4.109	2.35	High
Reback et al. (2021)	1.6	1.127	2.073	14.63	High
Wilson et al. (2021)	2.3	1.309	3.291	3.99	High
Morris et al. (2022)	1.6	0.862	2.338	6.87	Some
Jones et al. (2023)	2.2	1.626	2.774	10.69	High
Massa et al. (2023)	2.3	1.658	2.942	8.81	Some
Schnall et al. (2024)	1.4	0.789	2.011	9.6	High
Theta (θ)	1.817	1.613	2.021	–	

Table 2 presents the results of the heterogeneity analysis for the studies included in this meta-analysis, which assesses the degree of variation in effect sizes across individual studies and the potential impact on the reliability of the pooled estimate. Cochran's Q statistic was 12.58 (degrees of freedom = 10), with a *p*-value of 0.2481. Since this value exceeds the conventional significance level of 0.05, it suggests that the heterogeneity between the studies was not statistically significant. Therefore, the results across the studies were consistent.

**Table 2:** Heterogeneity analysis of relevant studies.

**Measure**	**Value**	***df***	***P*-value**
Cochran's *Q*	12.58	10	0.2481
H_2_	1.15	1	–
*I*^2^ (%)	13.32	0.0%	0.0%
*Z*	17.45	–	< 0.0001

The *H*^2^ value was 1.15, which is close to 1, further supporting the conclusion of low heterogeneity among the studies. The *I*^2^ statistic was 13.32%, which is well below the commonly accepted threshold of 50%. This indicates that only approximately 13% of the total variation in effect sizes can be attributed to genuine differences between the studies, with the remaining variation likely due to sampling error rather than substantial differences in the research.

In addition, the *Z*-test for the overall effect size was 17.45 (*p* < 0.0001), a highly significant result, confirming the robust efficacy of digital health interventions. Taken together, the low level of heterogeneity observed across these studies supports the appropriateness of using a random-effects model for the meta-analysis. The resulting pooled effect size demonstrated high internal consistency and credibility, reinforcing the stability of the conclusions drawn from the data.

**Figure 2:**
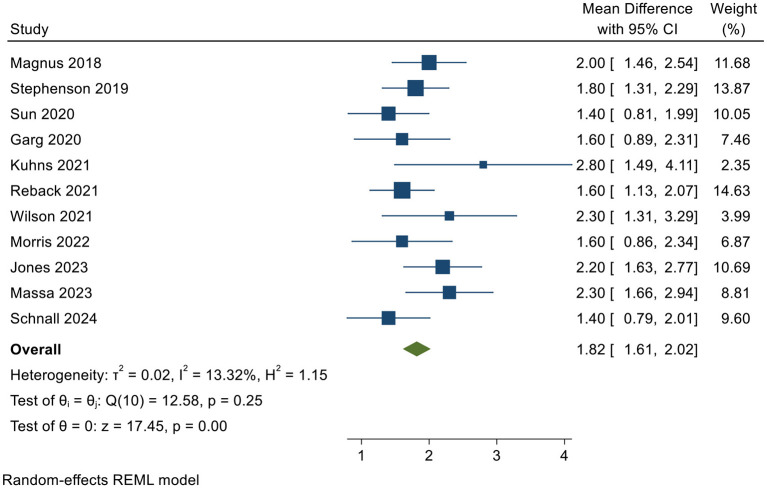
Forest plot.

Figure 2 visually presents the 11 individual studies included in this meta-analysis along with their corresponding pooled effect sizes. Each horizontal line represents the effect estimate and its 95% confidence interval for a specific study, with the size of the central block indicating the study's weight in the pooled analysis. All point estimates from the studies are positioned to the right of the null line (effect size = 1), and the confidence intervals for most studies did not include 1. This suggests that digital health interventions have a positive effect on enhancing HIV prevention and treatment adherence among transgender populations.

The study by Kuhns et al. (2021) reported the highest effect size (2.8), although its wide confidence interval and low weight (2.35%) suggest considerable uncertainty in the findings. In contrast, the studies with the highest weights, Reback et al. (2021) at 14.63% and Stephenson et al. (2019) at 13.87%, produced more stable effect size estimates with narrow confidence intervals, making them the most influential in determining the overall pooled results. At the bottom of the plot, the diamond represents the final random-effects pooled estimate (θ = 1.817), with a 95% confidence interval of 1.613–2.021. This confidence interval is entirely to the right of the null line and concentrated within a narrow range, further confirming the statistical significance and precision of the intervention effect.

The forest plot shows a relatively concentrated distribution of effect sizes, consistent with the low level of heterogeneity observed in the analysis (*I*^2^ = 13.32%). This reinforces the consistency and reliability of the research findings, indicating a robust conclusion regarding the efficacy of digital health interventions.

**Figure 3:**
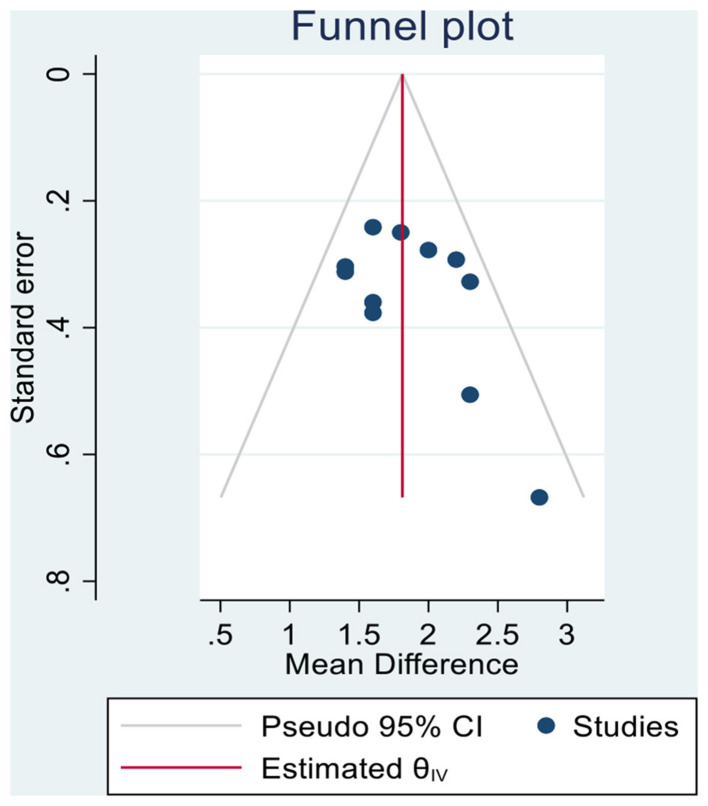
Funnel plots.

Funnel plots were used to evaluate the potential for publication bias or small-sample effects in this meta-analysis (Figure 3). These plots display the effect sizes of the individual studies on the horizontal axis and their standard errors on the vertical axis, typically inverted. In the absence of bias, study points should cluster symmetrically around the pooled effect size, typically represented by a vertical dashed line, forming an inverted funnel shape.

Upon visual inspection, the majority of study points are concentrated near the top of the funnel, where standard errors are smaller and precision is higher. This distribution is relatively symmetrical on both sides of the centerline, suggesting that the findings from large-sample, high-precision studies were balanced and consistent. While some points appear near the bottom of the funnel, potentially reflecting studies with smaller sample sizes or greater variability, their distribution does not show a pronounced unilateral clustering tendency.

Further supporting the absence of publication bias, Egger's regression test (β1 = 1.89, *p* = 0.147) indicated that the null hypothesis of “no small-sample effect” could not be rejected (*p* > 0.05). This suggests that publication bias was not a significant concern. Taken together, both the visual symmetry of the funnel plot and the quantitative test results indicate that publication bias had minimal influence on the pooled effect size, thereby enhancing the reliability and validity of the research conclusions.

**Figure 4:**
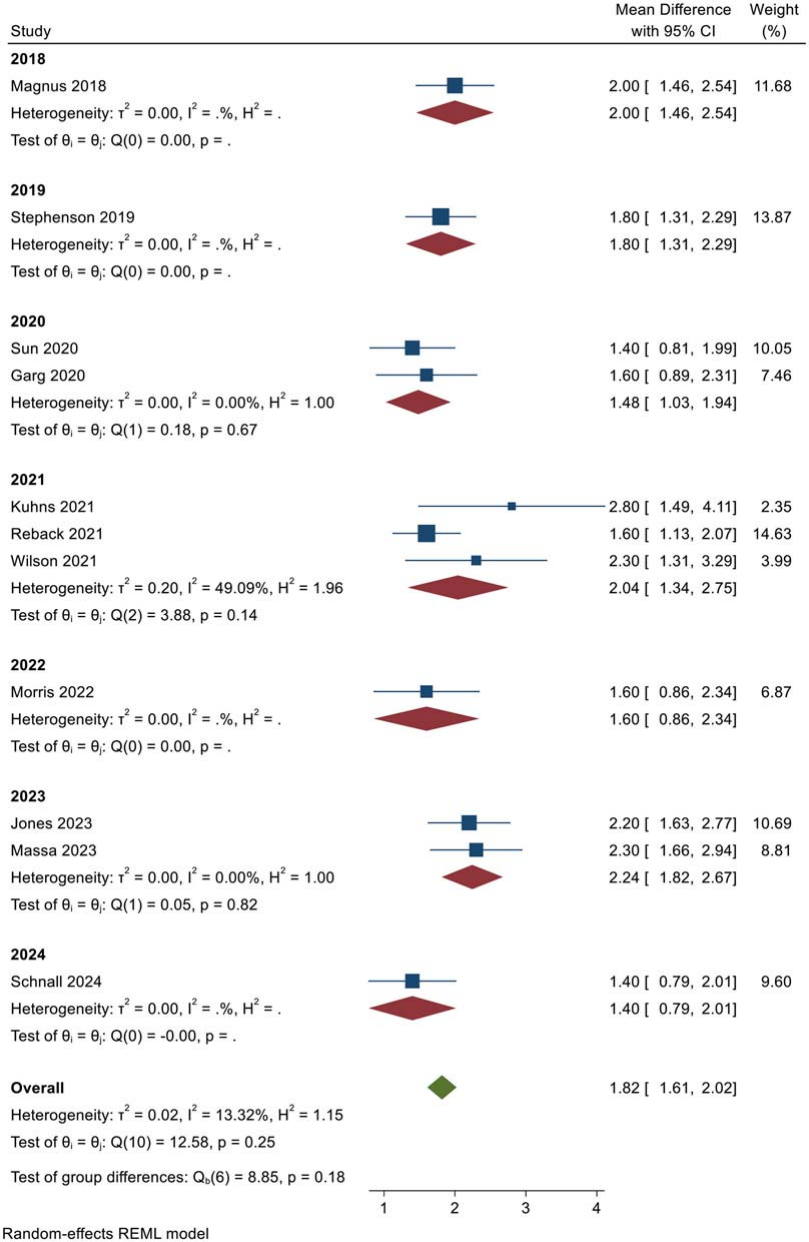
Subgroup analysis results.

Figure 4 presents the results of the meta-analysis grouped by year, aiming to explore temporal trends and intra-group consistency in the effectiveness of digital health interventions for transgender populations. The chart categorizes the included studies into seven time periods, ranging from 2018 to 2024, and calculates the pooled effect size and heterogeneity statistics for each subgroup.

Figure 4 provides insights into the effectiveness of digital health interventions across different years. Each subgroup represents a specific year, and the corresponding effect sizes along with their 95% confidence intervals are displayed for each group. In 2018, Magnus et al. (2018) reported an effect size of 2.00 (95% CI: 1.46–2.54), contributing 11.68% to the overall weight, with no heterogeneity observed (*Q* = 0.00, *p* = N/A). Similarly, in 2019, Stephenson et al. (2019) reported an effect size of 1.80 (95% CI: 1.31–2.29), contributing 13.87% to the overall weight, with no heterogeneity found (*Q* = 0.00, *p* = N/A). In 2020, the pooled effect size from studies by Sun et al. (2020) and Garg et al. (2020) was 1.48 (95% CI: 1.03–1.94), with heterogeneity statistics indicating no significant variation (*H*^2^ = 1.00, *Q* = 0.18, *p* = 0.67), confirming consistency within the subgroup.

In 2021, the pooled effect size from (Kuhns et al. 2021), (Reback et al. 2021), and (Wilson et al. 2021) was 2.04 (95% CI: 1.34–2.75), with Kuhns et al. (2021) reporting the highest effect size of 2.80. Despite some variability in effect sizes, the heterogeneity (*H*^2^ = 1.96, *Q* = 3.88, *p* = 0.14) was not significant, suggesting no major differences among the studies in this subgroup. Morris et al. (2022) reported an effect size of 1.60 (95% CI: 0.86–2.34) in 2022, contributing 6.87% to the overall weight, with no observed heterogeneity (*Q* = 0.00, *p* = N/A). In 2023, Jones et al. (2023) and Massa et al. (2023) yielded a pooled effect size of 2.24 (95% CI: 1.82–2.67), with no significant heterogeneity (*H*^2^ = 1.00, *Q* = 0.05, *p* = 0.82). Finally, Schnall et al. (2024) reported an effect size of 1.40 (95% CI: 0.79–2.01) in 2024, contributing 9.60% to the overall weight, with no heterogeneity observed (*Q* = 0.00, *p* = N/A). Overall, the analysis indicates consistent and significant intervention effects across all time periods, with no major heterogeneity across the years.

Table 3 presents the results of a statistical test for publication bias, specifically assessing small-sample effects, using Egger's linear regression method. The null hypothesis (H0) posited that β1 = 0, suggesting no small-sample effects. The test results showed a regression coefficient (β1) of 1.89, with a standard error (SE) of 1.305. The calculated *Z*-statistic was 1.45, yielding a *p*-value of 0.1470. Since this *p*-value exceeds the conventional significance level of 0.05, there is no evidence of a significant small-sample effect in this meta-analysis. This suggests that it is unlikely that publication bias influenced the results.

**Table 3:** Results of publication bias testing.

**Measure**	**Value**
**H0: beta1** = **0; no small-study effects**	
beta1	1.89
SE of beta1	1.305
*Z*	1.45
Prob > |*z*|	0.1470

## Discussion

### Acceptability and feasibility of digital platforms

The results of this meta-analysis provide compelling evidence that digital health interventions significantly enhance HIV prevention and treatment adherence among transgender populations. The pooled effect size (1.82) indicates that digital platforms have a meaningful and consistent impact across studies. The findings from the subgroup analysis further support this, as effect sizes in each time period exceeded 1, with narrow confidence intervals consistently excluding 1, indicating a statistically significant positive effect. Moreover, the relatively low heterogeneity across studies (*I*^2^ = 13.32%) suggests that the results are reliable and robust, enhancing confidence in the generalizability of these findings. These outcomes underscore the acceptability of digital platforms in improving health behaviors, as digital tools offer flexibility, anonymity, and convenience, all of which are essential for addressing the unique challenges faced by transgender individuals in accessing HIV-related care.

The high level of consistency across studies suggests that digital platforms are a feasible intervention. The studies included in the meta-analysis spanned multiple years and diverse research contexts, yet all demonstrated positive effects, indicating that digital health tools can be effectively implemented across various settings. The absence of significant publication bias (*p* = 0.1470) further suggested that the results were not unduly influenced by selective reporting or small-sample effects, reinforcing the feasibility of these interventions. Given the growing global demand for health interventions that are both scalable and adaptable, digital platforms appear to be a promising solution to the traditional barriers limiting healthcare access for transgender populations.

### Addressing stigma and psychosocial barriers

In addition to their acceptability and feasibility, digital health interventions are particularly effective in addressing the stigma and psychosocial barriers that often hinder transgender individuals' engagement with HIV prevention and treatment programs. The use of digital platforms provides a private and non-judgmental space for users, which is crucial for individuals who may experience discrimination in traditional healthcare settings. By offering anonymous or confidential interactions, these platforms allow transgender individuals to seek advice, support, and treatment without fear of stigmatization (Ayala et al., 2025; Lee, 2023; Keuroghlian et al., 2021; Ogunbajo et al., 2024).

The results of the subgroup analysis, which included studies from 2018 to 2024, show a consistently positive impact of digital health interventions across time, underscoring the growing recognition of the role these platforms can play in addressing psychosocial barriers. The positive effect sizes observed in 2020, 2021, and 2023 (ranging from 1.48 to 2.24) suggest the broad applicability of these interventions across different contexts, providing evidence that digital platforms can be tailored to meet the needs of diverse transgender populations.

Moreover, the relatively low heterogeneity across studies (*I*^2^ = 13.32%) suggests that the effectiveness of these interventions is not significantly influenced by the specific challenges or barriers encountered in different settings, further supporting their capacity to address the stigma and psychosocial barriers that transgender individuals face. In particular, studies with higher weights, such as those by Reback et al. (2021) and Stephenson et al. (2019), which demonstrated stable effect sizes and narrow confidence intervals, underscore the reliability and reproducibility of these findings across different contexts.

## Strengths

This systematic review and meta-analysis provides a comprehensive evaluation of DHIs in HIV prevention and care engagement among transgender individuals. The inclusion of 11 studies highlights the growing body of evidence on the effectiveness of digital health platforms, including mobile applications and telehealth interventions, across different contexts. The consistently positive effect sizes across these studies, with a pooled effect size of 1.82, suggest that digital health interventions are not only effective but also feasible and acceptable for improving health outcomes, such as HIV prevention and treatment adherence among transgender populations.

The individual effect sizes ranged from 1.4 to 2.8, and all confidence intervals excluded 1, further emphasizing the significant impact of these interventions. The low heterogeneity observed across studies (*I*^2^ = 13.32%) reinforces the robustness and consistency of these findings, suggesting that DHIs are effective across various settings and populations. In addition, the absence of publication bias, as confirmed by Egger's regression test (*p* = 0.1470), enhances the reliability of the results. Collectively, these findings demonstrate that DHIs can effectively support HIV prevention and care engagement while maintaining high user satisfaction, offering promising solutions to the barriers faced by transgender individuals in accessing HIV-related care.

## Limitations

Although this review provides strong evidence, several limitations must be considered. First, the heterogeneity of study designs, sample sizes, and assessed outcomes may introduce variability. However, the high homogeneity observed in the meta-analysis (*I*^2^ = 0.0%) suggests that these differences did not significantly affect the overall findings. A potential limitation is the unclear long-term impact of digital interventions, particularly regarding sustained engagement in HIV care and ART adherence. The absence of studies focusing on a wider range of transgender subgroups (e.g., transgender men and non-binary individuals) limits the generalizability of the findings. The majority of studies were conducted in high-income settings, which may affect the applicability of the findings in low- and middle-income countries where access to digital resources might be limited. In addition, the reliance on self-reported data in several studies raises concerns about reporting biases, especially when assessing sensitive behaviors such as HIV testing and care engagement.

## Study implications

This study has important implications for the development and implementation of digital health interventions aimed at transgender populations at risk for or living with HIV. The findings underscore the potential of digital tools to overcome traditional barriers to HIV care and prevention, such as stigma and geographical limitations. However, for DHIs to be truly effective, they must be designed with the unique needs of transgender individuals in mind, including gender-affirming care and mental health support. The high acceptability and feasibility of DHIs demonstrated in this study suggest that such interventions can play a key role in engaging transgender individuals in HIV care, but further research is needed to explore their long-term effectiveness, particularly in diverse geographical and socio-economic contexts. Moreover, greater inclusivity is needed to ensure that DHIs cater to a broad range of transgender individuals, including transgender men and non-binary persons.

## Conclusion

In conclusion, digital health interventions have shown promise as effective tools for enhancing HIV prevention and care engagement among transgender populations. However, variability in study designs, lack of long-term outcome data, and limited inclusivity of diverse transgender groups highlight the need for further research. To maximize the impact of digital health interventions on HIV prevention and care for transgender individuals, it is crucial to ensure that these interventions are culturally competent, accessible, and inclusive of the full spectrum of transgender identities. Continued innovation and evaluation will be essential to determine how these tools can be scaled and adapted to address the evolving needs of transgender communities worldwide.

## Data Availability

The original contributions presented in the study are included in the article/supplementary material, further inquiries can be directed to the corresponding author.
